# Quadratus lumborum block for total abdominal hysterectomy: a double-blind, randomized, controlled trial

**DOI:** 10.55730/1300-0144.5978

**Published:** 2025-02-07

**Authors:** Nada PEJCIC, Marija KUTLESIC, Ivan VELICKOVIC, Vladimir MILIC, Slavisa KOVACEVIC, Radmilo J. JANKOVIC, Dejan MITIC, Radomir MITIC, Nenad ZORNIC

**Affiliations:** 1 Department of Anesthesiology and Reanimatology, Leskovac General Hospital, Leskovac, Serbia; 2 Clinic for Anesthesiology and Intensive Therapy, Clinic for Gynecology and Obstetrics, University Clinical Center Nis, Nis, Serbia; 3 SUNY Downstate Health Science University, Brooklyn, NY, USA; 4 Clinic for Anesthesiology and Intensive Therapy, University Clinical Center Nis, Nis, Serbia; 5 Clinic for Gynecology and Obstetrics, University Clinical Center Nis, Nis, Serbia; 6 Department of Surgery, Faculty of Medical Sciences, University of Kragujevac, Serbia

**Keywords:** Quadratus lumborum block, abdominal hysterectomy, postoperative pain management

## Abstract

**Background/aim:**

The Quadratus lumborum block (QLB) is an interfascial block recommended for multimodal pain therapy after cesarean section in patients who cannot receive intrathecal morphine. We wanted to investigate whether QLB contributes to better analgesia after total abdominal hysterectomy (TAH). The study was registered on the website ClinicalTrials.gov (registration number NCT05765318).

**Materials and methods:**

Patients scheduled for TAH were randomized into two groups of 30 members each. The treatment group received QLB, while the control group did not undergo any block. The primary outcome was the amount of morphine during the first 12 postoperative h. Secondary outcomes included the total amount of morphine for 24 h, time to the first request for additional analgesia, pain intensity at rest and during activity, frequency of nausea and vomiting, and the degree of sedation at 2, 6, 12, and 24 h postoperatively.

**Results:**

Patients in the QLB group received significantly less morphine (4.13 mg) compared to the control group (9.73 mg) during the first 12 postoperative h (p < 0.001) and also during the first 24 h. The patients in the QLB group had longer time interval to the first breakthrough pain (7.87 h) compared to the control group (2.63 h) (p < 0.001), lower NRS scores at rest and during activity after 2, 12, and 24 h, and better satisfaction with provided pain relief during the first 24 h postoperatively.

**Conclusion:**

QLB reduces postoperative morphine use and postoperative pain intensity on the NRS scale, both at rest and during activity, as part of multimodal pain therapy for TAH.

## 1. Introduction

The quadratus lumborum block (QLB) is a regional analgesic technique, an interfascial plane block that involves a local anesthetic injection into the quadratus lumborum plane [1]. The European Society of Regional Anaesthesia and Pain Therapy (ESRA) [2] and the Society for Obstetric Anesthesiology and Perinatology (SOAP) [3] recommended QLB for post-Cesarean pain management in cases where intrathecal morphine cannot be used or for management of breakthrough pain. The purpose of the study was to assess whether the QLB could improve pain control following a hysterectomy. Hysterectomy is the second most common surgical procedure in the female reproductive system after cesarean section [4]. Moderate to severe pain persists beyond 3 months in 5 to 32% of patients who underwent open hysterectomy [5]. The procedure-specific postoperative pain management (PROSPECT) guidelines for abdominal hysterectomy published in 2006^1^ recommended laparoscopic or vaginal hysterectomy as a preferred surgical technique. Although the hysterectomy technique has shifted from an abdominal to a laparoscopic approach [6], a large number of patients still undergo total abdominal hysterectomy (TAH). Previous reports addressing the effects of QLB for laparoscopic hysterectomy are controversial [7, 8].

Inadequately treated acute postoperative pain is associated with delayed postoperative ambulation, an increased incidence of cardiovascular and respiratory complications, increased morbidity and mortality, and reduced patient satisfaction [5]. Persistent chronic postoperative pain is considered a major complication after surgery. It is related to the type of surgery, surgical technique, and perioperative pain management; it is difficult to treat and associated with serious psychosocial problems for the patient, as well as a decrease in work capacity [9, 10].

In Serbia, we have a significant percentage of TAH. We want to improve postoperative pain management in our clinical settings and reduce postoperative opioid consumption and its side effects.

The study aimed to assess whether QLB provides analgesic benefits in multimodal pain management after TAH in our clinical settings. We hypothesized that if the QLB were included in the postoperative pain management plan, it would have an additional analgesic effect, leading to an expected reduction in postoperative morphine consumption.

## 2. Materials and methods

### 2.1. Study design and setting

We designed a prospective, randomized, controlled study in accordance with the Declaration of Helsinki, approved by the institutional review board. The study was registered on the website ClinicalTrials.gov under the number NCT05765318. Written informed consent was obtained from all patients before inclusion, after explaining all the study details, including voluntary inclusion and data confidentiality. In total, 60 patients were included in the study from March 2023 to February 2024.

Inclusion criteria were the American Society of Anesthesiologists (ASA) physical status score of 1–3, age ≥ 18 years, body weight > 50 kg, and patients scheduled for elective TAH. Exclusion criteria included patient refusal to participate in the study, allergies to any study medication, local skin infection at the site of QLB injection, body mass index > 40 kg/m^2^, inability to comprehend or participate in scoring scales, difficult anatomy resulting in poor ultrasound visualization of muscular and fascial structures necessary for correct block administration, and daily regular intake of opioids or any other analgesics.

We used computer-generated randomization, preparing a list with six blocks of 10, resulting in a 1:1 intergroup ratio and ensuring equal distribution with a total of 30 subjects in each group. Sixty opaque sealed envelopes, numbered 1–60, were prepared. Patients were randomly allocated to either the QLB group (bilateral QLB performed after the end of surgery, during the emergence of anesthesia) or the control group (no block performed).

A study assistant, who was not involved in the anesthesia or postoperative monitoring of study participants, opened the sealed opaque envelope containing group allocation. If needed, three 20 mL syringes of bupivacaine 0.25% were prepared, and the anesthesiologist - block provider was informed. The envelopes were resealed by the two study assistants. The envelopes remained sealed until the completion of the study, including data sampling and statistical analysis of outcomes. As a result, investigators, participants, care providers, outcome assessors, and the statistician were all blinded to group allocation. If necessary, QLB was administered at the end of surgery, before emergence from anesthesia.

### 2.2. Anesthesia

Preanesthetic management started with intramuscular injection of midazolam at a dose of 0.07 mg/kg (maximum 7.5 mg) 30 min before induction, causing satisfying anxiolysis. During the surgery, the patients were monitored with 3-lead electrocardiography, pulse oximetry, noninvasive blood pressure, and end-expiratory CO_2_. Standardized general anesthesia includes induction with a propofol bolus of 1.5–2.5 mg/kg and rocuronium 0.6 mg/kg. Fentanyl 2.5 mcg/kg was given at induction, and repeated to keep the blood pressure and heart rate changes up to 20% of baseline. Dexamethasone 0.1 mg/kg was administered IV after induction of anesthesia as a part of multimodal analgesia. Sevoflurane in a 50% air/50% oxygen mixture with an end-tidal of 2.0 vol% was used as the maintenance agent. Mechanical ventilation was maintained to keep the end-expiratory CO_2_ values between 34 and 36 mmHg. An incremental dose of rocuronium (0.15 mg/kg) was repeated when needed. Ketoprofen 100 mg IV and metoclopramide 10 mg IV were administered 20 min before the end of surgery. At the end of the surgery, after partial recovery from neuromuscular blockade, patients received atropine 0.01 mg/kg and neostigmine 0.02 mg/kg before tracheal extubation. Patients were transferred to the post-anesthesia care unit (PACU) after extubation and adequate hemodynamic and respiratory recovery.

### 2.3. Block procedure

The bilateral posterior QLB (QLB2) [11] was performed under the guidance of a portable handheld ultrasound device (Butterfly Network Inc, Massachusetts, USA) set for musculoskeletal examination, using an isolated needle for peripheral nerve blocks with a length of 100 mm (B. Braun, Melsungen, Germany), while the patient was in the supine position. The target point is represented by the posterior border of the quadratus lumborum muscle (QLM) at the level of the termination of the internal oblique muscle (IOM). Local anesthetic was injected after perforating the thoracolumbar fascia behind the termination of the IOM. The thoracolumbar fascia provides specific elastic resistance to the pressure of the blunt peripheral nerve block needle. Its perforation is followed by dual confirmation, specific tactile sensation of tissue resistance loss, and visual ultrasound confirmation. After a negative aspiration test, injecting 1 mL of a local anesthetic solution provides the third confirmation of the desired needle tip localization, ultrasonically visible hydrodissection – a growing hypoechoic shadow of accumulated fluid that separates the muscle from the fascia. This is also confirmation of the extravascular localization of the needle tip. The local anesthetic is administered fractionally in boluses of 5 mL each, following a negative aspiration test, monitoring the spread of the hypoechoic shadow of the injected local anesthetic. The blocks for all patients were performed by the same anesthesiologist, the author of the study.

### 2.4. Surgery

The standard surgical approach involved TAH using a Pfannenstiel incision. Hysterectomies with bilateral salpingo-oophorectomies were performed. A surgical drain was routinely placed.

### 2.5. Postoperative pain management

All patients received postoperative intravenous ketoprofen 100 mg every 12 h and intravenous morphine as needed. Morphine was administered by the nurse upon the patient’s request if the pain intensity on the numeric rating scale (NRS) was greater than 3/10 (for intensity 4–6/10, intravenous morphine 3 mg; and for NRS ≥7/10, intravenous morphine 5 mg). Pain reevaluation was performed by the nurse 20 min after administering morphine. If the pain intensity was still greater than 3, an additional morphine bolus would be given using the same principle, and the pain assessment would be repeated after 20 min. The maximum allowed amount of morphine was 40 mg within 4 h.

### 2.6. Outcomes

The primary outcome was cumulated morphine consumption (mg) during the first 12 postoperative h. Secondary outcomes were: pain at rest (NRS 0–10), at mobilization (aNRS 0–10), morphine consumption (mg) at 24 h postoperatively, time to first morphine demand, postoperative nausea and vomiting (PONV) (yes/no), sedation (deep/light or absent), and patient satisfaction with provided analgesia.

### 2.7. Assessment of outcomes

After transferring the patient from the operating room to the PACU, noninvasive blood pressure, continuous three-lead ECG, and pulse oximetry were monitored for 24 h. Pain intensity, PONV, and the level of sedation were also monitored. Their assessment was conducted at predefined time points: immediately upon arrival in the PACU (T0), 2 h thereafter (T2), 6 h after arriving in the PACU (T6), 12 h after arriving in the PACU (T12), and 24 h after PACU admission (T24), as well as upon every request for additional analgesia. The time to the first breakthrough pain was defined as the time interval from T0 to the moment of the request for additional analgesia when the patient received morphine.

Pain intensity was assessed by a nurse using the Numeric Rating Scale (NRS) from 0 to 10, where 0 indicates complete absence of pain, and 10 represents the most intense imaginable pain. Pain intensity was evaluated at rest (NRS) and with activities (aNRS), such as when bending the knees or coughing. PONV scores were assessed by the 5-point scale in which 0 indicates neither nausea nor vomiting, 1–mild nausea without any treatment required, 2–nausea that can be resolved with antiemetics, 3–vomiting that can be resolved with antiemetics, and 4–indicates nausea or vomiting that does not respond to antiemetics [12]. Sedation was graded by the following sedation grading system (SGS): grade 0–deeply sedated and unresponsive; grade 1–sedated but responsive to light glabellar tap or loud voice; grade 2–sedated but responsive to normal voice; grade 3–awake and responding [13]. The degree of sedation grade 0 and 1 were classified as deep sedation, while the degree of sedation grade 2 and 3 were categorized as light sedation or absence of sedation. Patient satisfaction with achieved analgesia in the first 24 h postoperatively was evaluated 24 h after transfer to the PACU (T24). Patients expressed their satisfaction on a three-point scale from 0 to 2; 0–dissatisfied with achieved analgesia, 1–satisfied, 2–highly satisfied with achieved analgesia [14].

### 2.8. Statistics and sample size

The sample size was calculated using G*Power version 3.1.97 (Heinrich Heine University, Düsseldorf, Germany). The effect size was determined based on our unpublished data. To achieve a study power of 0.8 with a significance level (α) of 0.05 (two-sided), the required sample size was 26 patients per group. Accounting for a 15% dropout rate, the final sample size was set at 30 patients per group, 60 patients in total.

Data were analyzed using IBM SPSS (IBM Corp. Released 2012. IBM SPSS Statistics for Windows, Version 21.0. Armonk, NY: IBM Corp.). Normal distribution was tested using the Shapiro-Wilk test. Variables were presented as mean (SD), median (IQR), count (%), and range as appropriate. A comparison of normally distributed continuous data was performed using Student’s unpaired t-test. The Mann-Whitney test was used to compare non-Gaussian distributions, ranks, and scores. For binomial data, we applied the χ^2^ test or Fisher’s exact test as appropriate. We used log-rank tests to compare Kaplan-Meier plots for the duration of time until the first opioid request. All reported p-values are two-sided. There is a statistically significant difference between groups if p < 0.05.

## 3. Results

**Figure 1:**
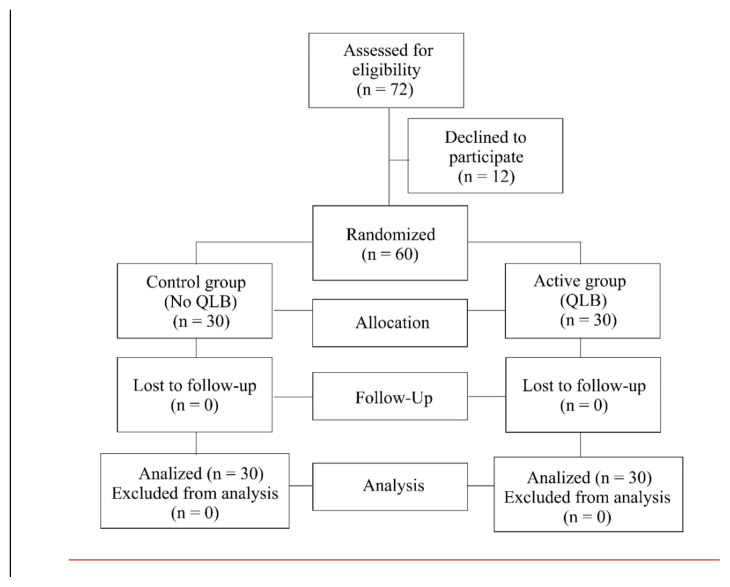
Consolidated Standards of Reporting Trials (CONSORT) flow diagram.

A total of 72 patients met the inclusion criteria for the study (Figure 1). Out of these, 60 agreed to participate in the study by signing the Informed Consent Form and were randomly assigned to either the active or control group, with 30 in each. No patient experienced perioperative adverse effects or complications that would warrant exclusion from the study. There were no differences in demographic characteristics, duration of the surgical procedure, or the amount of intraoperatively administered fentanyl between the study groups (Table 1). All patients underwent total abdominal hysterectomy with bilateral salpingo-oophorectomy through a Pfannenstiel incision.

During the first 12 h of the postoperative course, patients in the QLB group received an average of 4.13 (+/− 3.29) mg of morphine, which is approximately 5.6 mg of morphine (95% CI 3.44; 7.77) less than the control group without the block, who received an average of 9.73 (+/− 4.93) mg (Student’s T-test for independent samples, p < 0.001, the difference between the groups is statistically highly significant) (Table 2).

The patients who received QLB also required statistically significantly less morphine in the first 6 h (p < 0.001) and the first 24 h (p < 0.001) of the postoperative course compared to those who did not receive the block (Mann-Whitney U test for independent samples, a statistically highly significant difference, Table 2).

**Figure 2:**
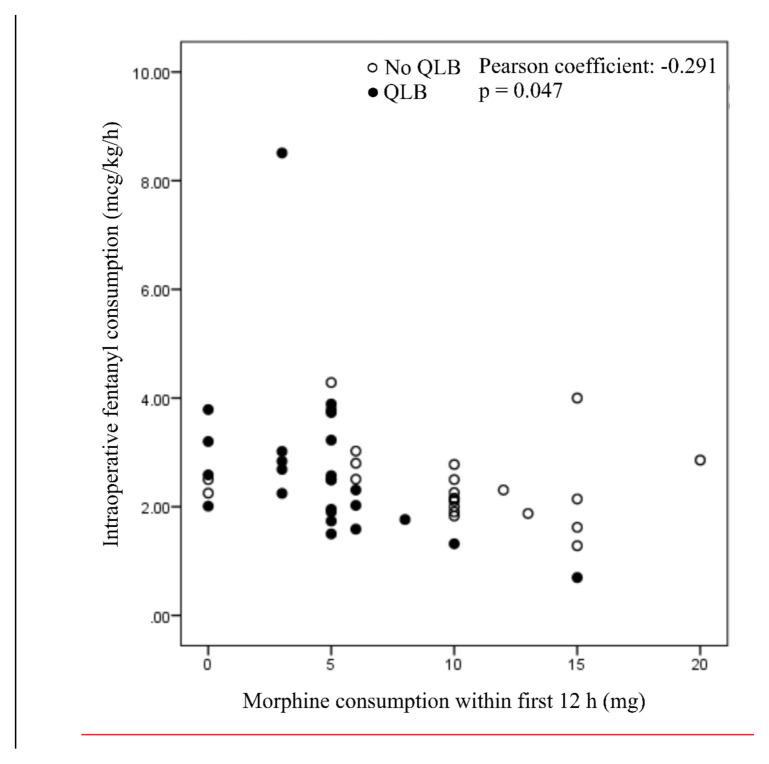
Correlation between intraoperative dose of fentanyl and postoperative morphine consumption. Scatter plot.

There is no difference in the intraoperative use of fentanyl between the groups (Table 1). There is a moderate negative correlation (Figure 2) between the amount of fentanyl administered intraoperatively and the amount of morphine administered postoperatively (Pearson coefficient is –0.291, p = 0.047, the correlation is statistically significant for p < 0.05). On the graphical representation (Figure 2), it is easily noticeable that patients in the QLB group received significantly less morphine than patients in the control group.

**Figure 3:**
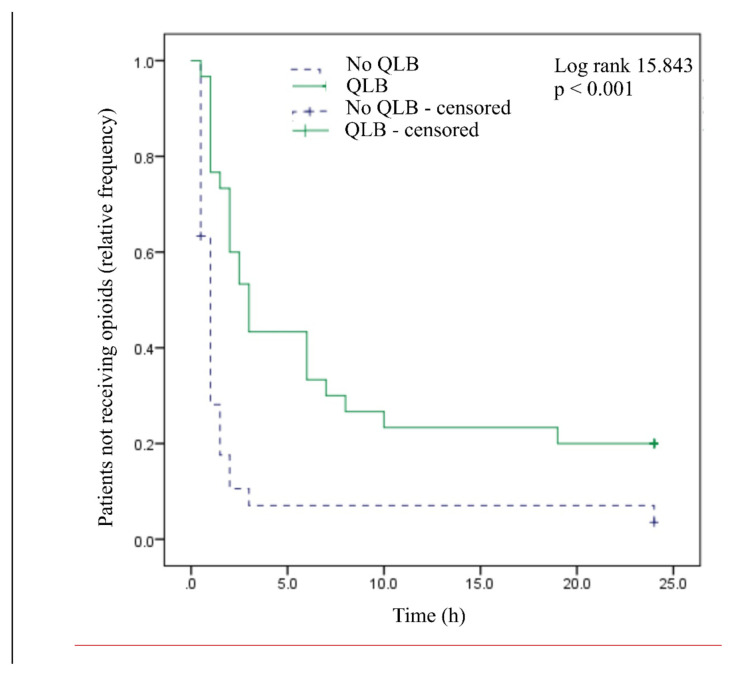
Time to first opioid request. Kaplan-Meier estimate of time (hours) to first opioid administration.

According to the Kaplan-Meier estimate, the time to first breakthrough pain is significantly longer in patients who received the block, 7.87 h (95% CI 4.70; 11.03), compared to those who did not receive it, where it averages 2.63 h (95% CI 0.4; 4.84) (Kaplan-Meier test, log-rank 15.843, p < 0.001; Figure 3).

A statistically significant lower NRS score at rest (Table 2) in the QLB group compared to the control group is observed at the following time points: T0 (p < 0.001), T2 (p < 0.001), T12 (p = 0.001), and T24 (p = 0.003). A statistically significant lower aNRS score during activity (Table 2) in the QLB group compared to the control group is observed at the following time points: T0 (p < 0.001), T2 (p < 0.001), T12 (p = 0.001), and T24 (p < 0.001) (Mann-Whitney U test for independent samples, p < 0.05 values are statistically significant, p < 0.001 values are statistically highly significant).

Although in all monitoring time intervals, PONV was more frequent in patients who did not receive QLB for postoperative pain therapy, a statistically significant difference in the frequency of PONV between the groups was demonstrated only after 12 h of stay in the PACU (Fisher’s test, p = 0.0257, p < 0.05 values are statistically significant; Table 3).

Patients who received QLB were more sedated upon admission to the PACU (p = 0.0016) and after 12 h from admission (p = 0.0022) compared to those who did not receive the block (Fisher’s test, statistically significant difference for p < 0.05; Table 4).

There were no differences in noninvasive blood pressure and heart rate between the groups during the 24-h postoperative monitoring period. The difference in blood oxygen saturation was present at the arrival in the PACU; patients who received QLB had significantly better saturation (Mann Whitney U test of independent samples, p = 0.009, values p < 0.05 are statistically significant).

Patients who received the block for postoperative analgesia were more satisfied with pain control during the first 24 h postoperatively compared to patients who did not receive the block (Fisher’s test, p = 0.033, statistically significant difference for p < 0.05; Table 5).

There were no complications associated with performing QLB (neither infections at the puncture site, hematomas, nor numbness/weakness in the legs).

## 4. Discussion

Patients who received QLB in multimodal postoperative pain therapy after TAH required a significantly lower amount of morphine compared to patients who did not receive the block at 2, 6, 12, and 24 h after admission to the PACU. QLB reduced morphine consumption by 50%. Lin et al. [15] and Korgvee et al. [16], in their meta-analyses, demonstrated a statistically significant reduction in morphine use in patients who received QLB compared to the control group without QLB (whether it was patients receiving a placebo block or not receiving a block at all) after 24 h; there was no difference in morphine consumption between the groups after 6 and 12 h. Lin et al. [15] included controlled randomized studies in their meta-analysis that followed the effects of posterior QLB in multimodal analgesia for various open abdominal interventions (abdominoplasty, inguinal hernia repair, cesarean section) and laparoscopic interventions (gynecological surgery, cholecystectomy, colectomy, gastrectomy, kidney surgery) in the adult population. Korgvee et al. [16] included observational studies in addition to randomized controlled studies in their meta-analysis, which followed the effect of QLB after abdominal laparoscopic surgery and hip surgery. Their conclusions regarding morphine consumption are nearly identical.

We observed significantly lower NRS and aNRS scores at the moment of arrival in the PACU, after 2, 12, and 24 h. The aNRS score is considered to be directly correlated with postoperative pulmonary complications [17]. Better pain control allows deeper breathing and more active patients, reducing the risk of lung congestion and respiratory complications. Supporting the improved pain control in our patients who received the block is the fact that they had better saturation upon arrival in the PACU, as they did not have to restrict breathing movements and could breathe more deeply. They were also more deeply sedated compared to patients who did not have the block upon arrival in the PACU, although there was no difference in the intraoperatively administered amount of fentanyl between the groups. Patients who received QLB were more deeply sedated even 12 h after leaving the operating room, despite receiving half the amount of morphine. This can also be attributed to better analgesia; well-pain-controlled patients could more easily drift into sleep. Lin and colleagues [15] demonstrated that posterior QLB significantly reduces NRS scores 6, 12, and 24 h after surgery performed under general anesthesia. A significantly lower aNRS score was noted only after 12 and 24 h. In patients operated under spinal anesthesia with intrathecal morphine, the use of QLB does not make a statistically significant difference in NRS scores [15].

Both meta-analyses emphasize that better analgesic effects are achieved with a block performed by lateral and anterior approaches compared to the posterior QLB that we conducted. Elsharkawy et al. [18] in their anatomical and CT study showed that the spread of local anesthetic into the paravertebral space is more predictable, and more likely if injected as posteromedial QLB or lower thoracic erector spinae plane block (ESPB) than posterolateral QLB. Therefore, it is expected that posterior QLB and ESPB performed in the lower thoracic segments have a similar analgesic effect, and at the same time more potent than lateral QLB. Blanco [11] points out that posterior QLB is a more superficial block than anterior QLB, allowing for better ultrasound visualization, and more precise identification of the interfascial space where the local anesthetic should be injected. Also, the needle tip is directed at the QLM, minimizing the risk of peritoneum and bowel perforation [11].

Korgivi et al. [19] concluded that QLB did not demonstrate inferiority compared to continuous epidural analgesia after radical cystectomy. Contrarily, for postoperative analgesia after open liver resection, lower opioid consumption, better analgesia, faster mobilization from bed, and quicker recovery of bowel function are observed in the group of patients with thoracic epidural analgesia [20]. However, performing thoracic epidurals may be contraindicated in patients with coagulation disorders or the ones on anticoagulant medications.

Like other authors [16, 21], we obtained results indicating a longer time to breakthrough pain in the group that received QLB. This period is longer both when compared to a placebo [11, 22] and when compared to a transversus abdominis (TAP) block in adults [23, 24] and children [25, 26]. Ipek [26] and Öksüz [27] demonstrated that in the pediatric population undergoing abdominal interventions, QLB proved to be more potent than the caudal block.

The study author has significant experience in performing both QLB and ESPB [27], and believes that QLB is much safer, especially in hospitals where ultrasound-guided regional anesthesia and analgesia techniques are newly introduced. ESPB is administered relatively close to the spinal cord and pleura, while QLB is performed far from vital organs. To perform ESPB, the patient must be in a sitting or lateral position, while QLB can be performed with the patient in a supine position under general anesthesia. All of these make QLB very attractive for beginners in interfascial block techniques.

QLB is considered a safe regional technique. Serious complications related to the performance of QLB have not been described so far. Since QLB is a classical intramuscular injection of medication, the risk of infection is much lower than that associated with peripheral nerve blocks or neuraxial blocks. An important advantage of QLB over anterior abdominal wall blocks (TAP, ilioinguinal-iliohypogastric block) is the fact that the needle pathway and the site of local anesthetic application are far removed from the peritoneal cavity, visceral organs in the abdomen, and major blood vessels minimizing the risk of their puncture. There is no data on neurological damage since the local anesthetic is not injected near a major nerve but into a space rich in small nerve endings. Therefore, it is generally accepted that QLB can be performed under both general and neuraxial anesthesia [1]. The main adverse effect of the block is numbness or weakness in the lower extremities [15]. Therefore, caution is necessary when the patient first gets up from the bed. The occurrence of intramuscular hematoma has been noted after performing QLB in two children who underwent full heparinization one hour after the block. The hematomas resolved after a few days without any residual effects [28]. Due to the deep localization of the injection site, which is inaccessible for compression, QLB is designated as a deep block. The consequences of the hematoma in the space around the QLM are negligible compared to the consequences that a potential hematoma could cause in the spinal or epidural space. Blocks involving the use of a large volume of local anesthetic are associated with an increased risk of developing local anesthetic systemic toxicity (LAST). QLB has been shown to carry a much lower risk of developing LAST than TAP blocks [29]. Some recommendations to reduce the risk of developing LAST include, in addition to proper dosing of local anesthetic based on dry body weight, adding adrenaline 5 mcg/mL to the local anesthetic solution [30]. A group of authors [31] prolonged the duration of postoperative analgesia by adding adrenaline to ropivacaine during QLB. In any case, it is always necessary to consider the possibility of developing LAST, and actively monitor patients for 30–45 minutes after performing the block.

Among our patients, there were no complications related to the use of QLB. We had a limitation related to assessing the motor strength of the legs. Our patients do not get out of bed in the first 12–18 hours of the postoperative period.

Recently published meta-analysis [32] showed that regional analgesic techniques (both single shot and continuous catheter techniques), applied as an analgesic supplement to general or neuraxial anesthesia, had nearly halved the risk of developing postoperative neurocognitive deficit. Additionally, this meta-analysis demonstrated that regional analgesic techniques significantly reduced the NRS score and the frequency of PONV. Within a broad range of regional analgesia techniques for patients undergoing major noncardiac surgical procedures, the QLB for laparoscopic radical gastrectomy was included in this meta-analysis.

There are some limitations to this study. First, we did not assess block success by evaluation of dermatomal anesthesia due to the risk of unblinding the patient and staff to block allocation. Second, we had nurse-administered analgesia instead of patient-controlled analgesia with opioid administration on breakthrough pain.

We have demonstrated that QLB significantly contributes to analgesia as part of multimodal pain therapy in open abdominal hysterectomy, reducing postoperative morphine use and postoperative pain intensity on the NRS scale, both at rest and during activity. The patients in the QLB group had a longer period until the development of breakthrough pain compared to the patients in the control group, before requesting additional analgesia. Given its contribution to better pain control, QLB has the potential to shorten the time until patients can mobilize after surgery.

## Figures and Tables

**Table 1:** Patient characteristics.

Patients	Control group (n = 30)	Active group (n = 30)	p-value
Age, mean (SD)	56.78 (11.13)	56.97 (11.43)	p = 0.950 1
Weight (kg), mean (SD)	72.24 (11.26)	72.20 (14.24)	p = 0.991 1
Body mass index (kg/m^2^), mean (SD)	25.98 (3.30)	26.87 (4.76)	p = 0.420 1
Duration of surgery (min), mean (SD)	94.04 (19.24)	102.24 (18.78)	p = 0.116 1
Fentanyl (mcg/kg/h), median [IQR]	2.28 [2.05, 2.79]	2.49 [1.83, 3.11]	p = 0.949 2
ASA physical status, n (%)			
I	4 (13.3)	5 (16.7)	p = 1.0 3
II	23 (76.7)	22 (73.3)	
III	3 (10.0)	3 (10.0)	

No statistically significant intergroup difference was observed for any of the variables.

1 Independent samples t-test,

2 Mann Whitney U test of independent samples,

3 Fisher-exact test, p < 0.05 statistically significant.

SD – standard deviation, IQR – interquartile range, ASA – American Society of Anesthesiologists.

**Table 2:** Primary and secondary outcomes – morphine consumption, NRS scores at rest and activity.

	Control group (n = 30)	Active group (n = 30)	p-value
**Primary outcome**			
Morphine consumption within the first 12 h (mg), mean (SD)	9.73 (4.93)	4.13 (3.29)	**p < 0.001** 1
**Secondary outcomes**			
Morphine (mg) consumption during a period			
T0–T2, median [IQR]	5 [3, 5]	0 [0, 3]	**p < 0.001** 2
T2–T6, median [IQR]	5 [3, 5]	0 [0, 3]	**p < 0.001** 2
T6–T12, median [IQR]	0 [0, 5]	0 [0, 3]	p = 0.378 2
T12–T24, median [IQR]	0 [0, 0]	0 [0, 0]	p = 0.633 2
T0–T6, median [IQR]	10 [5, 10]	3 [0, 5]	**p < 0.001** 2
T0–T24, mean (SD) and median [IQR]	10.17 (5.30)	5 [3, 5]	**p < 0.001** 2
NRS 0, median [IQR]	4 [2, 5]	0 [0, 1]	**p < 0.001** 2
NRS 2, median [IQR]	3 [2, 5]	1 [0, 3]	**p < 0.001** 2
NRS 6, median [IQR]	3[2, 4]	2 [1, 4]	p = 0.123 2
NRS 12, median [IQR]	2 [2, 3]	1 [1, 2]	**p = 0.001** 2
NRS 24, median [IQR]	2 [1, 2]	1 [1, 2]	**p = 0.003** 2
aNRS 0, median [IQR]	5 [3, 6]	1 [1, 2]	**p < 0.001** 2
aNRS 2, median [IQR]	4 [3, 6]	2 [1, 3]	**p < 0.001** 2
aNRS 6, median [IQR]	4 [3, 5]	3 [2, 5]	p = 0.145 2
aNRS 12, median [IQR]	3 [3, 5]	2 [1, 3]	**p = 0.001** 2
aNRS 24, median [IQR]	3 [3, 3]	2 [1, 3]	**p < 0.001** 2

1 Independent samples t-test,

2 Mann Whitney U test of independent samples, p < 0.05 statistically significant;

SD – standard deviation, IQR – interquartile range, NRS – numeric rating scale at rest, aNRS – numeric rating score at activity.

**Table 3:** Postoperative nausea and vomiting.

Time	No PONV (grade 0)		PONV (grade 1+2+3+4)		p-value 1
	Control group n (%)	Active group n (%)	Control group n (%)	Active group n (%)	
**T0**	20 (66.7)	22 (73.3)	10 (33.3)	8 (26.7)	p = 0.7787
**T2**	21 (70.0)	27 (90.0)	9 (30.0)	3 (10.0)	p = 0.1042
**T6**	23 (76.7)	26 (86.7)	7 (23.3)	4 (13.3)	p = 0.5062
**T12**	22 (73.3)	29 (96.4)	8 (26.7)	1 (3.3)	**p = 0.0257**
**T24**	26 (86.7)	29 (96.4)	4 (13.3)	1 (3.3)	p = 0.3533

1 Fisher-exact test, p < 0.05 statistically significant;

PONV–postoperative nausea and vomiting.

**Table 4:** Postoperative sedation.

Time	Deep sedation		Light sedation or absence of sedation		p-value 1
	(grade 0+1)		(grade 2+3)		
	Control group	QLB	Control group	Active group	
	n (%)	n (%)	n (%)	n (%)	
**T0**	7	20	23	10	**p = 0.0016**
	(23.3)	(66.6)	(76.7)	(33.4)	
**T2**	2	6	28	24	p = 0.2542
	(6.6)	(20.0)	(93.4)	(80.0)	
**T6**	2	5	28	25	p = 0.4238
	(6.6)	(16.7)	(93.4)	(83.3)	
**T12**	4	16	26	14	**p = 0.0022**
	(13.3)	(53.4)	(86.7)	(46.6)	
**T24**	0	1	30	29	p = 1
	0	(3.3)	(100.0)	(96.4)	

1 Fisher-exact test, p < 0.05 statistically significant.

**Table 5:** Patient satisfaction with provided analgesia during the first 24 hours postoperatively.

Satisfaction score	Control group (n = 30) n (%)	Active group (n = 30) n (%)	p-value 1
0–dissatisfied	6 (20)	1 (3.3)	p = 0.033
1–satisfied	18 (60)	15 (50.0)	
2–highly satisfied	6 (20)	14 (46.7)	

1 Fisher-exact test, p < 0.05 statistically significant.

## Footnotes

1 PROSPECT working group. European Society of Regional Anaesthesia and Pain Therapy (2006). PROSPECT recommendations for abdominal hysterectomy [online]. Website https://esraeurope.org/prospect/procedures/abdominal-hysterectomy-2006/summary-recommendations-2 [accessed 11/02/2023]
